# The role of *N*-glycans of HIV-1 gp41 in virus infectivity and susceptibility to the suppressive effects of carbohydrate-binding agents

**DOI:** 10.1186/s12977-014-0107-7

**Published:** 2014-12-11

**Authors:** Leen Mathys, Jan Balzarini

**Affiliations:** Rega Institute for Medical Research, KU Leuven, Minderbroedersstraat 10, B-3000 Leuven, Belgium

**Keywords:** Carbohydrate-binding agents (CBAs), HIV, Envelope gp41, *N-*glycans

## Abstract

**Background:**

Carbohydrate-binding agents (CBAs) are potent antiretroviral compounds that target the *N-*glycans on the HIV-1 envelope glycoproteins. The development of phenotypic resistance to CBAs by the virus is accompanied by the deletion of multiple *N-*linked glycans of the surface envelope glycoprotein gp120. Recently, also an *N-*glycan on the transmembrane envelope glycoprotein gp41 was shown to be deleted during CBA resistance development.

**Results:**

We generated HIV-1 mutants lacking gp41 *N-*glycans and determined the influence of these glycan deletions on the viral phenotype (infectivity, CD4 binding, envelope glycoprotein incorporation in the viral particle and on the transfected cell, virus capture by DC-SIGN^+^ cells and transmission of DC-SIGN-captured virions to CD4^+^ T-lymphocytes) and on the phenotypic susceptibility of HIV-1 to a selection of CBAs. It was shown that some gp41 *N-*glycans are crucial for the infectivity of the virus. In particular, lack of an intact N616 glycosylation site was shown to result in the loss of viral infectivity of several (i.e. the X4-tropic III_B_ and NL4.3 strains, and the X4/R5-tropic HE strain), but not all (i.e. the R5-tropic ADA strain) studied HIV-1 strains. In accordance, we found that the gp120 levels in the envelope of N616Q mutant gp41 strains NL4.3, III_B_ and HE were severely decreased. In contrast, N616Q gp41 mutant HIV-1_ADA_ contained gp120 levels similar to the gp120 levels in WT HIV-1_ADA_ virus. Concomitantly deleting multiple gp41 *N-*glycans was often highly detrimental for viral infectivity. Using surface plasmon resonance technology we showed that CBAs have a pronounced affinity for both gp120 and gp41. However, the antiviral activity of CBAs is not dependent on the concomitant presence of all gp41 glycans. Single gp41 glycan deletions had no marked effects on CBA susceptibility, whereas some combinations of two to three gp41 glycan-deletions had a minor effect on CBA activity.

**Conclusions:**

We revealed the importance of some gp41 *N*-linked glycans, in particular the N616 glycan which was shown to be absolutely indispensable for the infectivity potential of several virus strains. In addition, we demonstrated that the deletion of up to three gp41 *N*-linked glycans only slightly affected CBA susceptibility.

**Electronic supplementary material:**

The online version of this article (doi:10.1186/s12977-014-0107-7) contains supplementary material, which is available to authorized users.

## Background

The envelope of the human immunodeficiency virus (HIV) carries two glycoproteins: the transmembrane gp41 and the surface gp120, which are non*-*covalently associated and both highly glycosylated. Gp120 contains 18–31 *N-*linked glycans of which about 56-73% are suggested to be high-mannose-type glycans [1]. This is unusual since cellular glycoproteins carry virtually exclusively complex-type glycans [2]. The transmembrane gp41 contains 4–8 *N-*linked glycosylation sites and all 4–5 *N*-glycans on the ectodomain were suggested to be complex-type glycans [3]. Together, the unusual dense carbohydrate shield on the viral envelope prevents the immune system to efficiently recognize the underlying protein backbone and is therefore crucial for the successful escape of the virus from the host immune system [4-6]. Targeting the glycan shield is considered to be more challenging than targeting proteins, due to the high heterogeneity originating from glycosylation and the pronounced flexibility of the glycans. However, it has been shown that carbohydrate-binding agents (CBAs) (in particular those with a binding specificity towards high-mannose-type glycans) are able to inhibit HIV infection with a high efficiency and specificity (reviewed in [7]). Additionally, these CBAs have been shown to display a high genetic barrier, forcing the virus to delete multiple *N-*glycans in its envelope glycoproteins in order to obtain significant phenotypic drug resistance [8-14]. Interestingly, the deletion of these *N-*glycans has been shown to increase the immunogenicity of the viral particle [14,15]. Therefore, CBAs may not only offer direct antiviral effects but may also offer indirect benefits, by the induction of an enhanced immune response against the (mutant) virus.

In the process of gaining phenotypic CBA resistance, HIV accumulates mutations in *N-*glycosylation sites on the *Env* gene in the presence of escalating CBA concentrations. In order to achieve significant levels of resistance to CBAs, one or two single envelope *N*-glycan deletions are often not sufficient, whereas a combination of several *N-*glycan deletions is often required. It has been shown by us and others that most, if not all of these glycan deletions occur in gp120 [8-14]. However, we found at least one *N-*glycan deletion in gp41 of HIV-1_NL4.3_ which appeared under selection pressure by the combination of the plant lectin *Hippeastrum* hybrid agglutinin (HHA) and the carbohydrate-specific monoclonal antibody 2G12 [11]. It was shown that the deletion of this glycan on N674 in gp41 somewhat decreased the susceptibility of the virus to several CBAs including the plant lectins HHA and *Galanthus nivalis* agglutinin (GNA), the prokaryotic lectin actinohivin (AH) and the non*-*peptidic antibiotic pradimicin-S (PRM-S). However, the mutation N674D had appeared in the context of virus selected in the presence of CBAs (HHA+2G12), containing not only the N674D mutation in gp41 but also 3 additional *N*-glycosylation site mutations in gp120 (N160, N339 and N386 –according to HIV-1_HXB2_ amino acid numbering). Therefore, we wondered whether the sole deletion of an *N-*glycan in gp41 could influence the susceptibility of HIV-1 to CBAs, in the absence of glycan deletions in gp120, which is so far considered to be the main target of CBAs.

The gp41 *N*-glycans have already been studied by others. However, since many of these reports contradict each other [16-20], the exact role of the gp41 *N*-glycans for HIV infection is still unclear.

The gp41 *N-*glycans might potentially play a role in CBA susceptibility since it has been shown before that some CBAs (i.e. AH and PRM-S), are able to bind gp41 in the absence of gp120 [21,22].

In order to gain more insight in the potential role of gp41 *N-*linked glycans in the antiviral activity of CBAs, we generated virus strains lacking individual (and combinations of) *N-*linked glycans in gp41, using site-directed mutagenesis. These mutant viruses were examined for their viral phenotype (infectivity, CD4 binding, envelope glycoprotein expression on the virus particle and the transfected cell, DC-SIGN-mediated capture and transmission of captured virions to CD4^+^ T cells) and their sensitivity towards a selection of highly active CBAs. In addition, the binding kinetics of a selection of CBAs towards gp41 were determined using the surface plasmon resonance (SPR) technology, to identify gp41 as potential target for these compounds.

## Results

### Site-directed mutagenesis of *N-*glycosylation sites in HIV-1_NL4.3_

**Figure 1:**
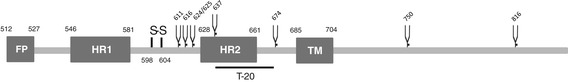
**Schematic representation of gp41 with indication of the** ***N-*** **linked glycosylation sites.** FP, fusion peptide; HR1, heptad repeat 1; HR2, heptad repeat 2; TM, transmembrane domain. Numbering was based on the HIV sequence compendium 2013 [23].

A selection of *N-*glycosylation sites in HIV-1_NL4.3_ gp41 was studied on their influence on viral function and susceptibility to the inhibitory effects of CBAs. It total, we could reveal 7 different *N-*glycosylation sites in HIV-1_NL4.3_ gp41, located on the asparagines 611, 616, 625, 637, 674, 750 and 816 (numbering of HIV-1_HXB2_ Env according to the HIV sequence compendium of 2013 [23]) (Figure 1). The glycosylation sites comprising N611, N616 and N625 are found in the linker region between heptad repeat (HR) 1 and 2. The glycosylation site containing N637 is located inside HR2, while N674 is positioned between HR2 and the transmembrane domain of gp41. The remaining two *N*-glycosylation motifs are found in the cytoplasmic tail of gp41 (N750 and N816).

Since the CBAs with pronounced anti-HIV activity are mostly high-molecular weight compounds which are not passing the cell membrane, we focused on *N-*glycosylation sites in the extracellular portion of gp41 and deleted these gp41 *N*-glycans using site-directed mutagenesis on N611, N616, N625, N637 and N674 by replacing the asparagine (N) by a glutamine (Q).

### Infectivity potential of wild-type and mutant HIV-1_NL4.3_ strains lacking individual gp41 *N*-glycans

In order to determine the importance of the individual *N-*linked glycosylation sites on gp41 for the viral infectivity of HIV-1_NL4.3_, CD4^+^ T-lymphocyte C8166 cells were infected using equal viral loads of wild-type (WT) or gp41 *N*-glycan*-*deleted virus strains.

**Figure 2:**
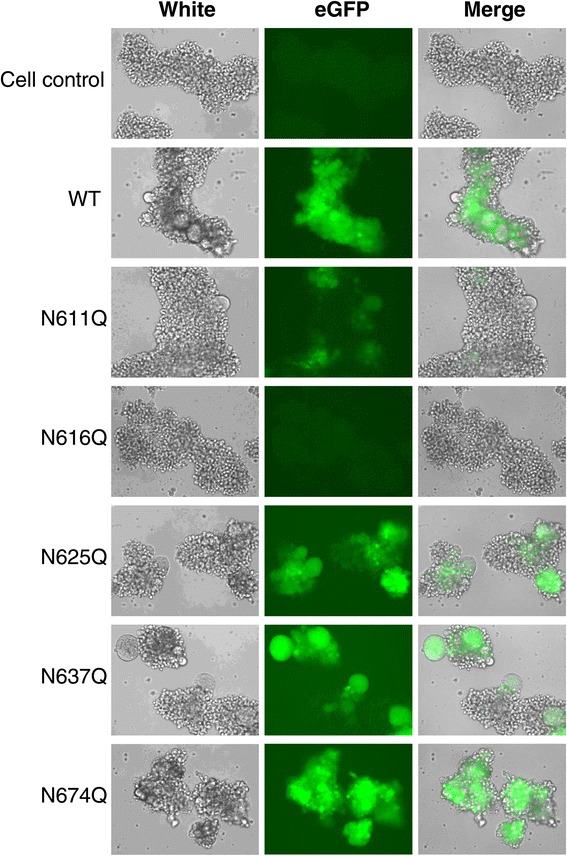
**Cytopathic effect of WT and mutant HIV-1**_**NL4.3**_ **on C8166 CD4** ^**+**^ **T cells.** Cells were infected with equal amounts of virus, based on the p24 capsid protein. Three days post infection, the (mutant) virus-infected cell cultures were inspected using light microscopy to visualize syncytia formation and fluorescence microscopy to quantify eGFP expression.

In a first experiment, virus was allowed to infect the cell cultures and to replicate during 3 days, after which the result of the viral infection (giant cell formation) was quantified using light microscopy. Additionally, the presence of the enhanced green fluorescent protein (eGFP) gene in the HIV-1_NL4.3_ virus used in the infection experiments enabled the determination of eGFP expression by fluorescence microscopy as a measurement of viral infection and replication. As is shown in Figure 2, infection of C8166 cells with WT virus resulted in the appearance of numerous giant cells and the abundant expression of eGFP. Based on the microscopic observations, no marked differences between infection with WT virus and infection with the gp41 glycan*-*deleted mutants N625Q, N637Q and N674Q gp41 HIV-1 strains could be observed. However, infection with mutant N611Q gp41 HIV-1 resulted in a marked decrease in both giant cell formation and eGFP expression. The gp41 mutant N616Q HIV-1 strain was seemingly without any viral infectivity potential.

**Figure 3:**
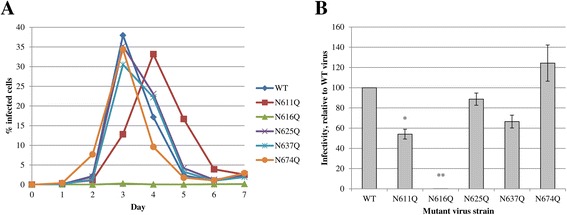
**Infectivity of WT and mutant gp41 HIV-1**_**NL4.3**_ **strains. A.** At day 0, CD4^+^ T-lymphocyte C8166 cells were infected with comparable viral loads, based on equal amounts of the p24 capsid protein. For 7 consecutive days, samples were harvested, fixed and analysed for eGFP expression by flow cytometry. **B.** The infectivity as presented in panel A was quantified using a linear regression to part of the curves, starting at day 1 post infection to the peak of the infectivity curve. Data are the means ± SEM of at least 5 independent experiments. The difference between WT and mutant virus was considered to be significant when the *p* value calculated using the student’s t-test was <0.05 (* = *p* < 0.05, ** = *p* < 0.005).

In a second experiment, the viral infectivity was quantified using flow cytometric determination of eGFP expression by the (mutant) virus-infected cells. Therefore, C8166 cells were infected and during 7 consecutive days, cell culture samples were harvested and analyzed. Figure 3A shows the results of one (out of 5) representative experiments, from which it became clear that there are differences in the infectivity of the WT and mutant HIV-1_NL4.3_ strains lacking one gp41 *N*-linked glycan. In order to obtain more quantitative data, the slope of the curves was calculated, starting from the data points at day 1 and ending at the top of the infectivity curves (Figure 3B). It was confirmed that the gp41 N616Q mutant HIV-1 lacked any viral infectivity. The gp41 HIV-1 mutants N611Q and N637Q also proved to have a decreased viral infectivity, which was only significant for mutant N611Q. The other mutants (N625Q and N674Q) had a viral infectivity not different from WT virus.

### Ability of WT and glycan*-*deleted HIV-1 strains to bind CD4

**Figure 4:**
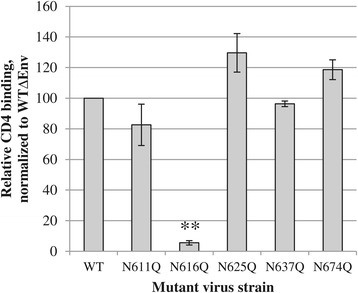
**Capacity of WT and mutant gp41 virus strains to bind to sCD4.** On average 48 ng p24 of the (mutant) HIV-1_NL4.3_ strains was lysed using triton-X100 and brought into contact with ELISA strips coated with sCD4. The binding efficiency was calculated after normalization to WT virus lacking envelope glycoprotein expression (to correct for aspecific interactions) and as relative to the binding of WT virus (set at 100%). Data are the means ± SEM of at least 2 to 4 independent experiments. The difference between WT and mutant was considered to be significant when the *p* value calculated using the student’s t-test was <0.05 (* = *p* < 0.05, ** = *p* < 0.005).

Given the fact that several mutant HIV-1_NL4.3_ strains lacking individual gp41 *N*-glycans had a decreased or even complete lack of viral infectivity, we wondered whether this could be due to a deficient binding to the primary receptor, CD4. Therefore, an enzyme-linked immunosorbent assay (ELISA) to quantify the binding efficiency of virus to soluble CD4 was performed (Figure 4). The results were normalized to WT virus lacking envelope glycoprotein expression (WT∆Env) to account for aspecific protein-protein interactions. It could be shown that the gp41 mutant N611Q, N637Q and N674Q HIV-1 strains had a binding efficiency to soluble CD4 (sCD4) that was not markedly different from WT virus. However, the gp41 mutant N625Q showed a non-significant increase (~130% of WT) in binding efficiency. The mutant N616Q, which was earlier shown to be uninfectious, was found to have a significantly decreased CD4 binding (~5% of WT). These findings may be explained by an altered gp120/gp41 conformation that does not efficiently recognize CD4 anymore, or, alternatively, that the envelope of the mutant gp41 N616Q virus virtually lacked glycoproteins in its envelope.

### Expression and incorporation of viral glycoproteins in WT and mutant HIV-1_NL4.3_ strains lacking one *N*-linked glycan in gp41

**Figure 5:**
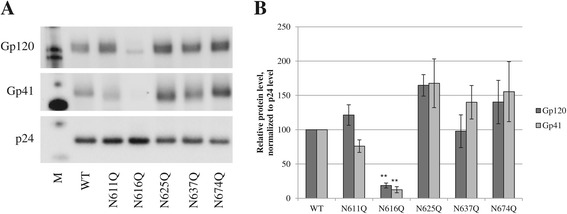
**Western blot analysis of expression and incorporation of gp120 and gp41 in the viral envelope of WT and mutant gp41 virus strains. A.** Analysis of the levels of gp120 and gp41 incorporation in virus particles. P24 was added to ascertain an equal-loading of the samples. M: MagicMark XP Western protein standard (Novex). All 3 panels are part of the same original blot (Additional file 1: Figure S1). **B.** Relative protein levels as quantified based on panel A using the ImageJ software, presented as normalized to p24 levels. Data are the means ± SEM of at least 5 independent experiments. The difference between WT and mutant virus was considered to be significant when the *p* value calculated using the student’s t-test was <0.05 (* = *p* < 0.05, ** = *p* < 0.005).

Given the lack of efficient CD4 binding by the mutant gp41 N616Q virus, and since *N-*glycosylation plays a crucial role during protein synthesis for correct envelope glycoprotein folding, we examined the levels of gp120 and gp41 in WT and mutant gp41 virus strains. Therefore, virus was lysed and analyzed by Western blot. Figure 5A visually shows that there is indeed variation in the level of incorporated gp120 and gp41 in the mutant gp41 viruses, especially for the uninfectious mutant gp41 N616Q virus which seems to have an almost complete lack of viral envelope glycoproteins. The three panels of Figure 5A originated from the same Western blot, shown in Additional file 1: Figure S1. The data in Figure 5A were then quantified and the levels of gp120 and gp41 were normalized to the p24 capsid protein levels (Figure 5B). It was shown that all mutants except for the N616Q mutant contained marked levels of gp120 and gp41 in their envelope that were not significantly different from the levels in WT virus. In contrast, the mutant gp41 N616Q HIV-1 was shown to have significantly decreased levels of gp120 and gp41 in its envelope.

### Flow cytometric analysis of gp120 incorporation in the cell membrane of transfected HEK293T cells

**Figure 6:**
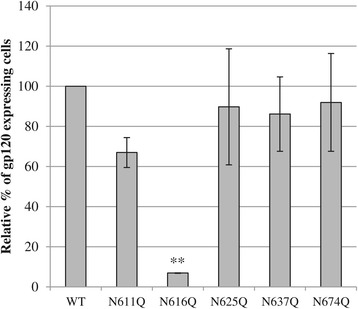
**Flow cytometric analysis of gp120 incorporation in the cell membrane of transfected HEK293T cells.** HEK293T cells were transfected to produce WT or gp41 mutant HIV-1_NL4.3_. After 3 days, gp120 on the cell surface was detected using the monoclonal antibody 2G12. The percentage of gp120-expressing cells was determined using flow cytometry. Data are the means ± SEM of 2 independent experiments. The difference between WT and mutant virus was considered to be significant when the *p* value calculated using the student’s t-test was <0.05 (* = *p* < 0.05, ** = *p* < 0.005).

In addition to the analysis of gp120 and gp41 levels in viral particles, we also investigated the incorporation of functional gp120 in the cell membrane. Therefore, HEK293T cells were transfected to produce WT or mutant gp41 HIV-1 particles. Gp120 on the cell membrane was detected using the monoclonal antibody 2G12 that recognizes a glycosylated epitope on natively folded gp120. As is shown in Figure 6 the deletion of glycan N616 in gp41 resulted in a highly decreased incorporation of gp120 in the cell membrane of the transfected HEK293T cells. The other glycan deletions in gp41 had no or only minor effects on gp120 incorporation. These data are in agreement with the western blot data on the gp120 levels in the virus particles (Figure 5).

### Efficiency of virus capture by Raji/DC-SIGN cells and efficiency of transmission of DC-SIGN-captured virions to C8166 cells

**Figure 7:**
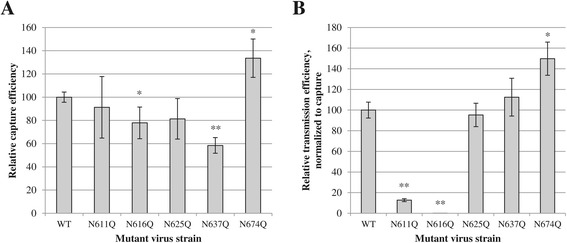
**Efficiency of virus capture by DC-SIGN** ^**+**^ **Raji cells and the subsequent transmission of captured virus to C8166 T cells. A.** Raji/DC-SIGN cells were exposed to virus during 1 h, after which unbound virions were removed by thourough washing. The levels of captured virus were quantified using a p24 ELISA and were used as a measurement for capture efficiency. All data were normalized to the capture efficiency of virus lacking envelope glycoprotein expression (WT∆Env). **B.** Virus-captured Raji/DC-SIGN cells were brought into contact with C8166 T cells, resulting in transmission of virus to, and infection of, the C8166 cells. Virion production by the C8166 cells, as quantified by p24 ELISA, was used as a measurement for transmission efficiency. Results represent transmission efficiency, normalized to equal levels of virus capture by the Raji/DC-SIGN cells. Results are means ± SEM of at least 3 independent experiments. The difference between WT and mutant gp41 virus was considered to be significant when the *p* value calculated using the student’s t-test was <0.05 (* = *p* < 0.05, ** = *p* < 0.005).

In a first set of experiments, WT and mutant gp41 virions (lacking individual gp41 *N*-glycans) were exposed to DC-SIGN^+^ Raji cells, allowing the binding (capturing) of DC-SIGN to gp120-containing virus. After elimination of unbound virions, the amount of captured virus was quantified using a p24 ELISA, as a measurement of the capture efficiency by DC-SIGN. To control for alternative interaction pathways, not involving the recognition of gp120/gp41 glycans by DC-SIGN, we included WT virus lacking envelope glycoproteins (WT∆Env). It was shown that this virus had a capture efficiency of ~40% as compared to WT virus (data not shown). We normalized all data to WT∆Env virus, to only take into account DC-SIGN-mediated capture of WT *versus* mutant virus particles. Figure 7A shows that the N611Q and N625Q mutant gp41 virus strains were endowed with a capture efficiency not significantly different from WT virus. The mutant gp41 N616Q and N637 viruses had statistically lower capture efficiencies of ~80% and ~60%, as compared to WT virus. In contrast, the mutant gp41 N674Q HIV-1 showed a ~30% increase in capture efficiency.

In a second set of experiments, virus-captured DC-SIGN^+^ Raji cells were brought into contact (co-cultured) with C8166 cells, resulting in the transmission of captured virions from the Raji/DC-SIGN cells to the C8166 cells. The latter cells will then be infected and subsequently produce new virus particles. The production of virus particles was quantified using a p24 ELISA and was used as a measurement for the transmission efficiency. Figure 7B shows that mutant virus strains containing the N625Q and N637Q mutations in gp41 had transmission efficiencies equal to WT virus. The mutant N674Q virus strain had an increased transmission efficiency, while the mutant N611Q showed a highly decreased transmission efficiency. The N616Q gp41 mutation resulted in a complete absence of virus transmission, which is consistent with the finding that this mutation was also highly detrimental on virus infectivity, CD4 binding and envelope glycoprotein expression. As expected, WT∆Env also lacked transmission potential (data not shown).

### Conservation of the gp41 *N-*glycosylation sites among several HIV-1 subtypes

**Figure 8:**
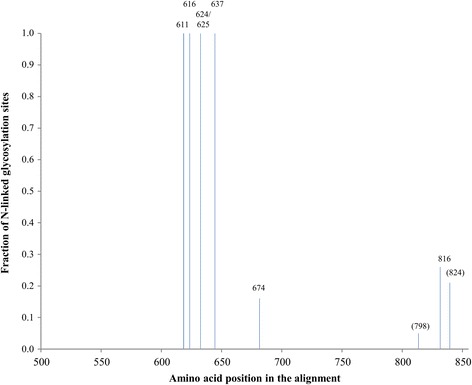
**Conservation of** ***N*** **-linked glycosylation sites in gp41 of several HIV subtypes.** An alignment of consensus sequences (2004) was obtained from the HIV sequence database [23] and was analysed using the program N-glycosite [23] to predict *N*-linked glycosylation sites in gp41. The alignment contained consensus sequences for HIV subtypes A1, A2, B, C, D, F1, F2, G, H, CRF01-AE, CRF02-AG, CRF03-AB, CRF04-cpx, CRF06-cpx, CRF08-BC, CRF10-CD, CRF11-cpx, CRF12-BF, and CRF14-BG. The consensus of consensus sequences and the ancestral sequences were excluded from the analysis. The fraction of HIV subtypes containing a glycosylation site at a specific location was determined. The positions (HXB2 numbering) of the glycosylated asparines are incidated on top of the lines. Glycosylation sites that were absent in HIV-1_NL4.3_ are indicated between parentheses.

We found that at least one gp41 glycan (N616) was indispensable for a wild-type-like viral phenotype of HIV-1_NL4.3_ (efficient cell-free infection, CD4 binding, envelope glycoprotein expression and transmission of DC-SIGN-captured virus to CD4^+^ T-lymphocytes), and the deletion of two other *N*-glycans (N611 and N637) also showed decreased infectivity of the corresponding mutant HIV-1_NL4.3_ strains. Therefore, the conservation of these glycosylation sites among different HIV-1 subtypes was examined, which would confirm their importance for the survival of the virus. We aligned consensus sequences of gp41 of >200 different HIV-1 strains belonging to 19 different subtypes and found that the first 4 N-terminal gp41 glycosylation sites (N611, N616, N624/N625 and N637) are highly conserved (Figure 8). The glycosylation site at position N674 was present in 3 out of 19 subtypes (A2, CRF04-cpx, CRF14-BG). The *N*-glycosylation site involving residue N798 was only found in HIV-1 subtype F1. The glycosylation site containing residue N816 was found in 5 out of the 19 sequences, from subtypes B, F1, H, CRF11-cpx and CRF12-BF. The glycosylation site of residue N824 was found in the sequences of HIV-1 subtypes G, CRF02-AG, CRF06-cpx and CRF14-BG. This analysis confirms the importance of the glycosylation sites involving residues N611, N616 and N637. Additionally, the glycan on asparagine 624/625 is 100% conserved and may therefore also be endowed with an indispensable function for the virus. The remaining 4 glycosylation sites, of which only two (N674 and N816) are present in HIV-1_NL4.3_, are less conserved among the analyzed HIV-1 subtypes.

In the initial search for *N-*glycosylation sites in HIV-1_NL4.3_ Env we found an additional *N-*glycosylation site involving N750. This glycosylation motif was not shown in the alignment presented in Figure 8. Taking a closer look on the HIV 2013 sequence of the Env protein compendium, we found that this glycosylation site is only present in 2 out of the approximately 200 HIV-1 strains included in this compendium. Therefore, we assume that this *N-*linked glycosylation site is not essential for the phenotype of HIV-1.

### The effect of the N616Q glycan deletion in gp41 on the infectivity and gp120 envelope levels of various HIV-1 strains

Deleting the N616 glycan in gp41 of HIV-1_NL4.3_ was shown to result in a highly significant decrease in viral infectivity (Figure 3). We also investigated the role of this *N*-linked glycan in gp41 of other HIV-1 strains. Therefore, we generated additional N616Q gp41 mutant virus strains (i.e. III_B_, ADA and HE). HIV-1_IIIB_ is, as NL4.3, a laboratory adapted HIV strain with a CXCR4 coreceptor tropism. The laboratory-adapted ADA strain uses CCR5 as coreceptor. HE is a clinical isolate of a Belgian HIV-1 patient. This virus strain is able to use both coreceptors during HIV entry.

**Figure 9:**
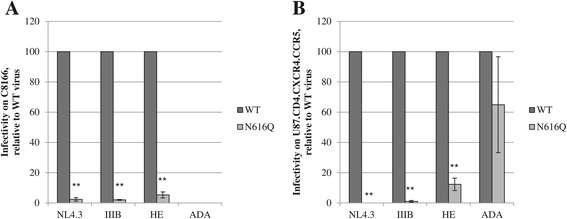
**Infectivity of various HIV-1 strains containing the gp41 N616Q glycan deletion.** C8166 **(A)** and U87.CD4.CXCR4.CCR5 **(B)** cells were infected with WT and mutant gp41 virus particles of strains NL4.3, III_B_, HE and ADA. After 3 and 5 days respectively, viral infectivity was determined using flow cytometric detection of eGFP expression in the infected cells. The HIV-1_HXB2_ amino acid numbering was used (N616Q), which corresponds to N614Q in HIV-1_NL4.3_, N616Q in HIV-1_IIIB_, N603Q in HIV-1_HE_ and N628Q in HIV-1_ADA_. Data are the means ± SEM of 2 independent experiments. The difference between WT and mutant gp41 virus was considered to be significant when the *p* value calculated using the student’s t-test was <0.05 (* = *p* < 0.05, ** = *p* < 0.005).

The gp41 N616Q mutant viruses were compared with their WT counterparts to investigate the effect of this glycan deletion on viral infectivity. Therefore, C8166 cells and U87.CD4.CXCR4.CCR5 cells were infected with WT and mutant virus. C8166 cells express CD4 and CXCR4 and are therefore susceptible to infection by NL4.3, III_B_ and HE. U87.CD4.CXCR4.CCR5 cells express CD4, CCR5 and CXCR4 and are susceptible to infection by all investigated virus strains. The infection efficiency was evaluated after 3 and 5 days respectively by determining the percentage of eGFP expressing cells using flow cytometry (Figure 9). It was shown that the deletion of the N616Q glycan resulted in a severe decrease in viral infectivity of HIV-1_NL4.3_, HIV-1_IIIB_ and HIV-1_HE_ in C8166 cells (Figure 9A). These data were confirmed using U87.CD4.CXCR4.CCR5 cells (Figure 9B). In contrast, it was shown that the N616Q mutation in HIV-1_ADA_ had much more modest suppressive effects on viral infectivity (not significant).

**Figure 10:**
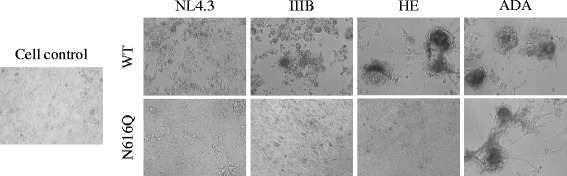
**The cytopathic effect of WT and mutant N616Q gp41 virus strains.** U87.CD4.CXCR4.CCR5 cells were infected with WT and mutant gp41 N616Q viruses of strains NL4.3, III_B_, HE and ADA. The cytopathic effect was evaluated after 5 days, using light microscopy.

The infectivity of the mutant virus strains was also evaluated based on their cytopathic effects on a U87.CD4.CXCR4.CCR5 monolayer cell culture. As shown in Figure 10, all WT viruses cause a cytopathic effect in the monolayer while most mutant viruses do not visibly affect the architecture of the monolayer, except N616Q HIV-1_ADA_ that was able to cause cytopathic effects similar to WT HIV-1_ADA_, which is in agreement with the flow cytometry results (Figure 9B).

**Figure 11:**
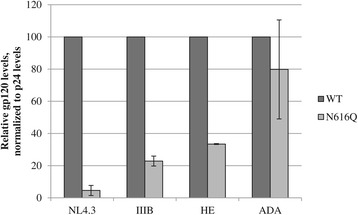
**Analysis of gp120 levels in the viral envelope of WT and mutant N616Q gp41 virus strains.** Relative protein levels based on quantification of western blot data using the ImageJ software, and presented as normalized to p24 levels. Data are the means ± SD of 2 independent experiments.

Based on the observation that HIV-1_NL4.3_ containing the gp41 N616Q glycan deletion had a severely decreased viral infectivity (Figures 2, 3, 9) due to significantly decreased levels of gp120 and gp41 in the viral envelope (Figure 5), we studied the gp120 levels in the other WT and N616Q mutant HIV-1 strains. As shown in Figure 11, severely decreased gp120 levels were found for mutant N616Q gp41 HIV-1 strains NL4.3, III_B_ and HE. This is in agreement with the decreased viral infectivities observed for these mutant virus strains, as compared to their WT counterparts. In contrast, the N616Q gp41 mutant HIV-1_ADA_ showed gp120 levels that were comparable to the levels observed in WT HIV-1_ADA_. This explains why the N616Q glycan deletion in gp41 of HIV-1_ADA_ did not result in a loss of viral infectivity.

### Binding kinetics of CBAs against gp120 and gp41

**Figure 12:**
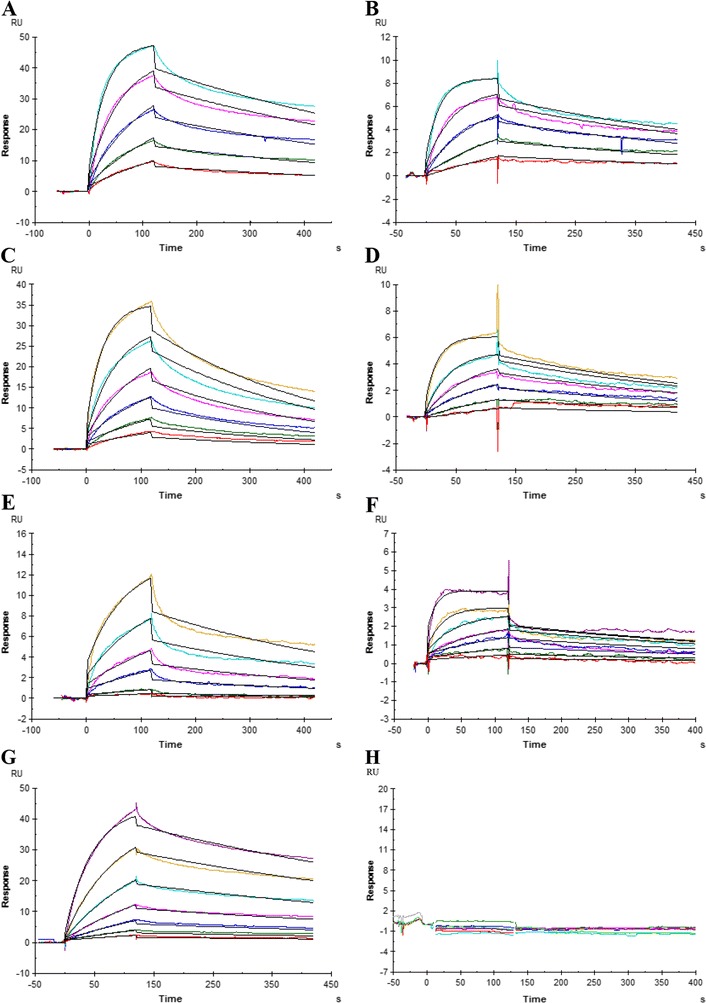
**SPR sensorgrams showing the binding and dissociation of CBAs to gp120 and gp41.** For each compound, a two-fold dilution series was tested and shown in different colors. The 1:1 binding model was used to fit the curves (shown in black). **A.** HHA vs gp120, highest concentration: 10 nM. **B.** HHA vs gp41, highest concentration: 10 nM. **C.** UDA vs gp120, highest concentration: 80 nM. **D.** UDA vs gp41, highest concentration: 80 nM. **E.** AH vs gp120, highest concentration: 80 nM. **F.** AH vs gp41, highest concentration: 80 nM. **G.** 2G12 vs gp120, highest concentration: 500 nM. **H.** 2G12 vs gp41, highest concentration: 500 nM. The results represent one of at least 2 independent experiments.

It could be that gp41 glycans are involved in the interaction of CBAs with the viral particle, since long-term exposure of virus to CBAs has led to the deletion of most of the glycans in gp120 and the glycan N674 in gp41 [11]. In order to determine whether CBAs are able to target gp41, we used the SPR technology to study the interaction of some of the CBAs (HHA, *Urtica dioica* agglutinin (UDA), AH, 2G12) *versus* gp120 and gp41, which were both covalently immobilized on a CM4 sensorchip. It was shown that HHA, UDA, AH and 2G12 were able to bind gp120 in a concentration dependent manner (Figure 12, left panels A, C, E and G, respectively). HHA, UDA and AH were also able to efficiently bind gp41 in a concentration dependent manner, while 2G12 failed to show a significant binding to gp41 (Figure 12, right panels B, D, F and H, respectively). A 1:1 binding model (suggesting the interaction of 1 ligand to 1 analyte) was used to fit the obtained sensorgrams and resulted in the determination of dissociation constants listed in Table 1. It was shown that all compounds bound to gp120 with a dissociation constant (K_D_) in the low nM range. The binding of the compounds to gp41 was also shown to have a K_D_ in the low nM range, except for 2G12 which was not able to bind gp41 as already mentioned above. The affinity of HHA to bind gp120 was about 1.5 times higher than the affinity towards gp41. For UDA, the difference in binding to gp120 vs gp41 was a factor 2.4. AH bound 6.8 times better to gp120 than to gp41.

**Table 1:** **Kinetic data for the binding of CBAs to gp120 and gp41**

	**Binding to gp120 (K**_**D**_ **in nM)**	**Binding to gp41 (K**_**D**_ **in nM)**	**Ratio binding to gp120/gp41**
**HHA**	0.5 ± 0.1	0.4 ± 0.1	1.5
**UDA**	9.0 ± 1.4	3.7 ± 0.3	2.4
**AH**	14 ± 1	2.0 ± 0.8	6.8
**2G12**	34 ± 6	No binding	/

Average ± SD, based on at least 2 independent experiments.

In conclusion, the carbohydrate-binding compounds HHA, UDA and AH were able to bind gp41 with K_D_’s in the nanomolar range, although with a somewhat lower affinity compared to binding to gp120. In contrast, the monoclonal carbohydrate-specific antibody 2G12 solely bound gp120 and showed a complete lack of affinity towards gp41.

### CBA susceptibility of WT and mutant gp41 virus strains lacking individual gp41 glycans

After confirming the ability of some CBAs to bind gp41 in the SPR assay, we investigated the sensitivity of gp41 glycan*-*deleted HIV-1_NL4.3_ strains to CBAs to reveal the influence of the lack of individual gp41 glycans on the interaction between CBAs and the viral particle. We made a selection of CBAs from different origins and carbohydrate specificities (HHA, UDA, AH, 2G12 and PRM-S) and, in addition, we also examined the mutant virus sensitivity to the fusion inhibitor enfuvirtide (T-20) and the CXCR4 antagonist AMD3100. The compounds were investigated for their antiviral activity towards WT and mutant (glycan-deleted) gp41 HIV strains in a cell-free virus infection assay. All compounds were highly active against WT HIV-1_NL4.3_, with EC_50_ values in the low nanomolar (HHA, AH, 2G12, AMD3100), median nanomolar (UDA), high nanomolar (T-20), or low micromolar (PRM-S) range (Table 2). Based on these results, we could conclude that none of the single gp41 *N*-glycan-deletions had a significant effect on the eventual antiviral activity of these compounds. In fact, only the mutant gp41 N637Q virus strain proved most affected by the test compounds, but this was consistently the case against all compounds, including, beside CBAs, also the bicyclam CXCR4 antagonist AMD3100 and the gp41-binding agent T-20. This mutation is located in the HR2 domain of gp41, which is an important amino acid stretch during the fusion process.

**Table 2:** **Inhibitory potential of a variety of HIV-1 inhibitors towards WT and mutant virus strains containing one** ***N*** **-glycan deletion in gp41**

	**HHA (nM)**	**UDA (nM)**	**AH (nM)**	**2G12 (nM)**	**PRM-S (** **μM)**	**T-20 (nM)**	**AMD3100 (nM)**
**WT**	0.9 ± 0.2	25 ± 3	5.2 ± 1.0	1.1 ± 0.2	4.8 ± 0.4	340 ± 141	3.5 ± 0.6
**N611Q**	1.6 ± 0.3	32 ± 6	5.4 ± 0.2	0.9 ± 0.2	5.3 ± 0.1	247 ± 177	2.5 ± 0.7
**N625Q**	1.4 ± 0.3	27 ± 5	8.1 ± 1.2	1.3 ± 0.2	4.5 ± 0.8	702 ± 255	2.9 ± 1.1
**N637Q**	3.7 ± 1.4	57 ± 24	15 ± 8	3.0 ± 2.0	5.3 ± 0.4	664 ± 454	7.6 ± 5.0
**N674Q**	1.3 ± 0.4	32 ± 11	8.3 ± 0.8	1.9 ± 0.5	5.2 ± 0.2	1530 ± 1201	3.8 ± 1.5

Values represent the EC_50_’s (50% effective concentration) of the test compounds expressed in nM or μM.

Data are the means ± SEM of at least 2 to 3 independent experiments.

### The effect of the N674D mutation in HIV-1 gp41

For the generation of glycan-deleted HIV-1 strains, we decided to alter the glycosylated asparagine into the structurally related glutamine, thereby eliminating glycosylation with a predicted minimal influence on other protein characteristics such as folding. However, during the selection of HHA/2G12 resistant virus we obtained a gp41 *N*-glycan deletion due to the N674D mutation, altering the asparagine into an aspartic acid. To determine the possible effect of this glycan deletion, we generated a mutant HIV-1_NL4.3_ strain containing the N674D mutation in gp41 and investigated the effect of this mutation on viral infectivity and CBA susceptibility.

**Figure 13:**
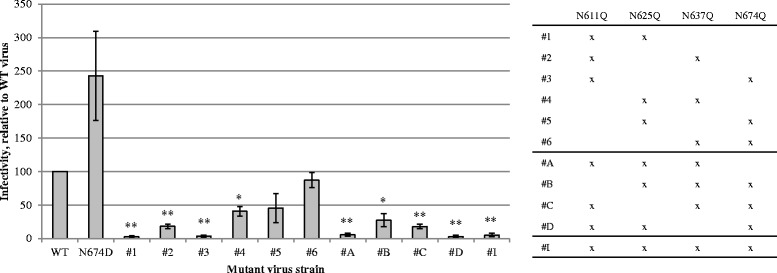
**Infectivity of HIV-1**_**NL4.3**_ **mutants containing the N674D mutation or a combination of gp41 glycan deletions.** In addition to the single glycan-deleting N → Q mutations in gp41, we also generated one N → D mutation and several combinations of N → Q mutations. The infectivity of these virus strains was studied using C8166 cells, which were infected at day 0. Subsequently, samples were harvested during 7 consecutive days. The number of eGFP positive cells was quantified using flow cytometry. The infection curves were used for linear regression analysis, to quantifiy the infectivity of the virus strains. Data are the means ± SEM of 2–3 independent experiments. The difference between WT and mutant virus was considered to be significant when the *p* value calculated using the student’s t-test was <0.05 (* = *p* < 0.05, ** = *p* < 0.005).

The N674D gp41 mutation resulted in an increased viral infectivity (although not significant) (Figure 13). In Figure 3, similar results were obtained for the N674Q mutant, which also showed a non-significant increase in viral infectivity.

The N674D mutation in gp41 was shown to have minor effects on CBA susceptibility (Table 3). It was shown that this mutation resulted in a 2-fold resistance towards HHA and an increased susceptibility towards AH (2.5-fold) and T-20 (5.4-fold). These results differ somewhat from the results obtained with the N674Q gp41 HIV-1 mutant (Table 2). Regarding AH, 2G12 and T-20, these mutants act opposite. While the N674Q gp41 HIV-1 mutant was slightly phenotypically resistant towards these compounds, the N674D mutation rather increased the susceptibility of the mutant virus towards these compounds.

**Table 3:** **Inhibitory potential of a variety of HIV-1 inhibitors towards WT and mutant virus strains containing the N674D mutation or a combination of glycan deletions in gp41**

	**Nr**	**HHA (nM)**	**UDA (nM)**	**AH (nM)**	**2G12 (nM)**	**PRM-S (** **μM)**	**T-20 (nM)**	**AMD3100 (nM)**
**WT**		0.9 ± 0.2	25 ± 3	5.2 ± 1.0	1.1 ± 0.2	4.8 ± 0.4	340 ± 141	3.5 ± 0.6
**N674D**		1.9 ± 0.6	34 ± 10	2.1 ± 0.5	0.8 ± 0.2	4.7 ± 0.4	63 ± 20	3.3 ± 0.3
**N611Q/N637Q**	#2	2.7 ± 0.4	63 ± 7	3.0 ± 0.7	0.5 ± 0.1	4.8 ± 0.4	49 ± 4	3.5 ± 0.3
**N625Q/N637Q**	#4	1.4 ± 0.2	51 ± 6	3.8 ± 0.1	0.8 ± 0.2	5.3 ± 0.1	21 ± 2	3.3 ± 0.4
**N625Q/N674Q**	#5	1.3 ± 0.3	30 ± 2	3.2 ± 0.3	0.9 ± 0.1	5.3 ± 0.1	57 ± 4	3.5 ± 0.1
**N637Q/N674Q**	#6	1.6 ± 0.5	28 ± 10	4.3 ± 0.9	0.9 ± 0.0	5.3 ± 0.1	48 ± 24	3.6 ± 0.3
**N625Q/N637Q/N674Q**	#B	2.1 ± 0.1	35 ± 6	3.2 ± 0.7	0.5 ± 0.1	5.0 ± 0.1	20 ± 2	3.5 ± 0.3
**N611Q/N637Q/N674Q**	#C	2.8 ± 0.6	52 ± 7	3.6 ± 0.4	0.6 ± 0.0	5.3 ± 0.2	56 ± 10	3.3 ± 0.2

Values represent the EC_50_’s (50% effective concentration) of the test compounds expressed in nM or μM.

Data are the means ± SEM of at least 3 independent experiments.

### The effect of multiple gp41 glycan deletions on viral infectivity and CBA susceptibility

We demonstrated that one single gp41 *N*-glycan deletion in HIV-1 could have detrimental effects on viral infectivity and that infectious mutant virus strains did not show a markedly increased or decreased susceptibility towards CBAs. However, pronounced CBA resistance generally only occurs after the concomitant deletion of several gp120 *N*-linked glycans. Therefore, we wondered whether CBA sensitivity could be altered by deleting more than 1 gp41 *N*-glycan.

Combinations of 2, 3 and 4 *N*-glycan deletions in gp41 were generated in HIV-1_NL4.3_. The infectivity of these virus strains was investigated using C8166 cells. As can be seen in Figure 13, many of the combined *N*-glycan deletions in gp41 led to a severe loss in viral infectivity, depending on the nature of the deleted *N*-glycans. No combinations were made involving the N616Q glycan in this study since this would most likely result in uninfectious virus strains.

The mutant virus strains with sufficient residual viral infectivity were investigated for their susceptibility towards CBAs (Table 3). Some of the mutant virus strains showed a minor decrease in susceptibility towards HHA and UDA, although these effects were limited. The maximal level of resistance observed was 3.1 for HHA (strain N611Q/N637Q/N674Q) and 2.5 for UDA (strain N611Q/N637Q). No susceptibility changes towards the non-peptidic antibiotic PRM-S and the CXCR4 antagonist AMD3100 were observed. For AH and 2G12 , minor increased susceptibilities were found (up to 2.5-fold for AH, 2.3-fold for 2G12). It was interesting to observe that all mutant gp41 HIV-1 strains with double and triple *N*-glycan deletions were markedly more sensitive to the inhibitory activity of T-20 (up to 17-fold) (Table 3). These data demonstrate that the antiviral activity of CBAs can slightly alter due to the absence of multiple gp41 *N*-glycans, however, this effect is only minor.

## Discussion

As is the case for all known antiretroviral drug classes, HIV tries to escape from the CBA inhibition pressure by gaining drug resistance. Up till recently, solely *N-*glycan deletions in gp120 were described to appear under CBA pressure and *N*-glycan mutations in the ectodomain of gp41 have never been described. However, we observed the deletion of the gp41 glycan at N674 during the selection of mutant HIV-1_NL4.3_ exposed to the combination of HHA and 2G12 in cell culture [11]. In the current study, we aimed to gain more insight in the role of gp41 *N-*linked glycans in the infectivity properties of HIV-1 and the susceptibility of such mutant virus strains to CBAs.

A single gp41 *N-*glycan deletion in HIV-1_NL4.3_ was unable to provoke significant phenotypical resistance towards the CBAs, although the deletion of some of the gp41 glycans had a significant effect on the viral infectivity. We demonstrated that the intact glycosylation site involving residue N616 of gp41 of HIV-1_NL4.3_ is absolutely indispensable for the correct expression of the envelope glycoproteins, the efficient binding to the primary receptor CD4, the viral infectivity and transmission of DC-SIGN-captured virus particles to CD4^+^ T-lymphocytes. In fact, a highly decreased level of gp120/gp41 was detected in the mutant gp41 N616Q HIV-1_NL4.3_ particle, resulting in a virtually complete loss of sCD4 binding and thus complete lack of infectivity/transmission potential when administered to CD4^+^ T-lymphocyte cell cultures. Beside the gp41 N616 deletion, we also found that deletion of the N611 and N637 glycans had a somewhat suppressive effect on viral infectivity, which was however markedly less pronounced than in the case of the gp41 N616 glycan deletion, and, opposite to the gp41 N616 mutant virus, the gp41 N611 and N637 mutant viral strains had normal CD4-binding properties and envelope glycoprotein levels.

We confirmed the importance of the N616 *N*-glycan of HIV-1_NL4.3_ gp41 in two other viral strains (HIV-1_IIIB_ and HIV-1_HE_). Strains III_B_ and NL4.3 are both laboratory-adapted HIV-1 strains that use the secondary CXCR4 receptor during virus entry, while strain HE is a clinical isolate that can use both CXCR4 and CCR5 for entry. However, the N616 glycan was found to be less crucial for the infectivity of the CCR5-using laboratory-adapted HIV-1_ADA_ strain. The loss of viral infectivity for the mutant HIV-1 strains NL4.3, III_B_ and HE could be correlated to severely decreased gp120 levels in their viral envelopes. In contrast, the gp120 level in the envelope of mutant N616Q gp41 HIV-1_ADA_ did not significantly differ from gp120 levels in WT HIV-1_ADA_. Therefore, we believe that the decreased viral infectivity seen in the mutant N616Q gp41 strains NL4.3, III_B_ and HE was caused by a compromised biosynthesis or trafficking of the envelope glycoproteins due to the N616Q glycan deletion in gp41. In this respect, it should be noticed that mutant R5-tropic N616A gp41 HIV-1 strains JR-CSF, JR-FL and BG505 kept pronounced infectivity [24]. Whether these findings might suggest that the N616 glycan in gp41 has a more important role for envelope glycoprotein synthesis and/or trafficking with CXCR4-tropic viruses than CCR5-tropic viruses need further investigation. However, when comparing our results with earlier published data on the gp41 *N*-linked glycans it seems that contradicting findings are reported. Lee *et al*. [16] showed in 1992 that the N637 glycan was crucial for viral infection using the HXB2 strain, which was cloned from the III_B_ isolate. They also found a decreased viral infection when the N616 glycosylation site was mutated. However, it was postulated that this was rather due to the nature of the amino acid change instead of the N616 glycan deletion [16]. The same year, Dedera *et al*. [17] found that both the N611 and N616 glycans were crucial for syncytium formation and viral infection of HXB2. In addition, they also found that the N637 glycan was important for viral infection [17]. Dash *et al*. [20] published in 1994 that the N637 glycan of gp41 was the most important glycan for viral function, using a virus strain (pEVd1443) that is related to the NL4.3 strain. Deleting this glycan was shown to result in a deficient envelope glycoprotein expression [20]. In 1998, it was shown by Perrin *et al*. that the N616 glycan was crucial for the infectivity of the X4 strain BH10 [19]. Recently, Wang *et al*. demonstrated that the presence of an intact N616 glycan was important for the infectivity of the FE strain, while this glycan deletion had no effect on the infectivity of strains YU-2 and Sc19-15 [18]. In conclusion, the gp41 glycans at positions N611, N616 and N637 have all been shown to be important for viral infectivity, but the predominant *N*-glycan seems to be dependent on the viral strain or the employed experimental procedures. Although we found the N616 glycan to be the most important glycan in gp41 for viral infectivity, we also found a decreased viral infectivity in case of mutations N611Q and N637Q as reported by several other researchers.

Mutating the gp41 N616 glycosylation site did only moderately reduce the capture efficiency by Raji/DC-SIGN cells (~20%), although reducing the envelope glycoprotein levels by ~85%. Similar results were obtained for at least one gp120 glycan-deletion (N156Q mutation in the V1/V2 region), where gp120/gp41 levels were reduced by 75% in the virus particle whereas the capture efficiency was unaffected (unpublished observations). We therefore may hypothesize that the interaction between DC-SIGN and gp120 is so highly efficient that even a highly reduced level of envelope glycoprotein expression (up to 85%) can still result in a capture efficiency comparable to WT virus. Alternatively, it is also possible that there is too much gp120 present in the HIV particle to be bound by a limited number of DC-SIGN molecules. A decrease in envelope glycoprotein expression would then only result in a severely compromised virus capture when the remaining levels of gp120/gp41 in the envelope are at least >85% lower than those observed in WT virus particles.

The N674D mutation that appeared in virus strains from the selection experiments using a combination of HHA and 2G12 could possibly be a rescue mutation. It was found that HHA/2G12-resistant virus suffered from a severe decrease in viral infectivity [11]. However, here we show that the N674D glycan deletion led to an increase (though non*-*significant) in the infectivity of HIV-1_NL4.3_. We therefore cannot exclude that the appearance of the N674D mutation in a background of gp120 mutations was merely a secondary mutation aiming to increase the infectivity of the resistant virus strain again, instead of a mutation that was directly the consequence of targeted pressure by HHA/2G12 and meant to increase the drug resistance level.

Our data also demonstrate that deletion of gp41 *N-*linked glycans did not significantly influence the susceptibility of HIV-1_NL4.3_ to CBAs. As earlier shown for gp120 by SPR technology, we now also showed that the CBAs were able to markedly bind to gp41, indicating that gp41 can be efficiently targeted by CBAs. However, on the virus particle, gp120 masks gp41 and may therefore hinder the interaction of CBAs with gp41. Only after gp120 has been bound to its CD4 receptor, conformational changes in gp120 induce the transient exposure of gp41. This could explain why, although CBAs are able to bind gp41, they are only rarely found to induce gp41 *N-*glycan deletions under long-term drug selection pressure. Thus, whereas gp41 might be a target for CBAs in HIV-infected cell cultures, these agents probably do not efficiently reach gp41 for interaction because the entry process of the virus is already blocked by the interaction of the CBAs with gp120. However, only in those cases where the virus can escape this CBA gp120-interaction blockade, the CBAs can eventually block the second CBA-susceptible step in the fusion process as well. This is probably the very first class of drugs that may efficiently and independently interact with two subsequent steps in the virus infection process, making them potentially very efficient drug candidates.

## Conclusion

We revealed the importance of some of the gp41 *N-*linked glycans, in particular at position N616. It was shown that the N616Q mutation in gp41 resulted in severely decreased gp120 levels in the viral envelopes of HIV_NL4.3_, HIV-1_IIIB_ and HIV-1_HE_, afforded markedly decreased CD4-binding by HIV-1_NL4.3_ and significantly decreased the infectivity of these viral strains. In addition we showed that, although CBAs have a pronounced affinity for gp41 glycans, their main primary target for viral inhibition is most likely gp120 rather than gp41.

## Methods

### Compounds

The high-mannose binding plant lectin GNA, derived from *Galanthus nivalis*, and the *N-*acetylglucosamine-specific plant lectin UDA, derived from *Urtica dioica*, were kindly provided by Prof. E. Van Damme (Ghent, Belgium). AH derived from the actinomycete K97-0003 was provided by H. Tanaka (Fukushima, Japan). The monoclonal antibody 2G12 was purchased from Polymun Scientific (Vienna, Austria). PRM-S was obtained from Prof. T. Oki and Prof. Y. Igarashi (Toyama, Japan). T-20 was kindly provided by the AIDS Research Alliance (Los Angeles, CA). AMD3100 was a kind gift of Dr. Gary Bridger (AnorMED, Langley, Canada) or obtained from Sigma-Aldrich (St. Louis, MO).

### Cells

Human CD4^+^ T lymphocytic C8166 cells were obtained from the American Type Culture Collection (ATCC) (Manassas, VA). Human Raji/DC-SIGN B-lymphocytic cells, expressing DC-SIGN, were constructed by Geijtenbeek *et al.* [25] and were kindly provided by Dr. L. Burleigh (Institut Pasteur, Paris, France). Both cell lines were grown in RPMI-1640 medium (Invitrogen, Merelbeke, Belgium), supplemented with 10% fetal calf serum (FCS) (Sigma, Bornem, Belgium), 2 mM L-glutamine and 2% gentamicin (Invitrogen).

Human embryonal kidney cells (HEK293T) were obtained from ATCC and were grown in Dulbecco’s Modified Eagle Medium (DMEM) (Invitrogen), supplemented with 10% FCS (Sigma), 75 mM NaHCO_3_ and 2% gentamicin (Invitrogen).

Microglial U87.CD4.CXCR4.CCR5 cells were provided by Professor D. Schols (Leuven, Belgium) and their construction and characterization are described elsewhere [26]. These cells were grown in DMEM supplemented with 10% FCS (Sigma), 75 mM NaHCO_3_, 0.002% gentamicin (Invitrogen), 0.0001% puromycin (Invitrogen) and 0.02% geneticin (Invitrogen).

HeLa-*tat*-III cells were obtained through the NIH AIDS Reagent Program, Division of AIDS, NIAID, NIH: HeLa-tat-III were originally constructed by Drs. William Haseltine, Ernest Terwilliger and Joseph Sodroski [27,28]. The cells were grown in DMEM medium (Invitrogen), supplemented with 10% FCS (Sigma), 75 mM NaHCO_3_, 2% gentamicin and 0.02% geneticin (Invitrogen).

### Plasmids

The construct pNL4.3_∆Env_eGFP was kindly provided by Dr. M.E. Quiñones‐Mateu (Lerner Research Institute, Cleveland, OH) [29]. This construct encodes all HIV-1_NL4.3_ genes except the gp160 envelope gene. In addition, this construct also encodes eGFP.

The construct pNLHIVx∆U∆ss, was a kind gift from Alenka Jejcic (at that time at the Karolinska Institute, Stockholm, Sweden) and allows the production of HIV-1_NL4.3_ particles lacking envelope glycoprotein expression.

The plasmid pBlue_Env(NL4.3) was used to amplify the gp160 gene of HIV-1_NL4.3_ for transfection purposes and was used for the cloning of pBlue_Env(III_B_), pBlue_Env(ADA) and pBlue_env(HE). Therefore, viral RNA was harvested for strains III_B_, ADA and HE using the QIAamp Viral RNA mini kit (Qiagen). The gp160 genes were amplified using the primers EnvB_EcoRI and HIV8726R_XhoI (Additional file 2: Table S1) and subsequently inserted in the pBlue backbone, to replace the NL4.3 gp160 gene.

### Viruses

HIV-1 was produced as described previously [30]. Briefly, HEK293T cells were cotransfected with a DNA construct encoding WT or mutant gp160 and XbaI-linearized pNL4.3_∆Env_eGFP. This leads to homologous recombination, thereby inserting the gp160 gene in the pNL4.3_eGFP backbone. Therefore, the HIV-1-infected cells express eGFP. The WT and mutant gp160 genes of four HIV-1 strains (NL4.3, III_B_, ADA and HE) were used to generate recombinant viruses. In this way, recombinant HIV-1_NL4.3_ was generated, as well as hybrid virus strains containing all NL4.3 genes except for the gp160 envelope gene which originated from HIV-1_IIIB_, HIV-1_ADA_ or HIV-1_HE_.

HIV-1_NL4.3_ lacking envelope glycoprotein expression was produced by transfecting HeLa-*tat*-III cells with the construct pNLHIVx∆U∆ss. Transfection was performed using GeneCellIn (BioCellChallenge, Nivelles, Belgium), according the instructions of the manufacturer.

### Site-directed mutagenesis

Mutations were introduced in the HIV-1 gp160-encoding plasmids pBlue_Env(NL4.3), pBlue_Env(III_B_), pBlue_Env(ADA), and pBlue_Env(HE). Therefore, we used the Quikchange Site-Directed Mutagenesis Kit (Agilent Technologies, Diegem, Belgium) and the primers listed in Additional file 2: Table S1. Plasmid DNA was purified using the PureLink Quick Plasmid Miniprep Kit (Invitrogen) and sequenced with the ABI PRISM BigDye Terminator v3.1 Ready Reaction Cycle Sequencing Kit (Applied Biosystems, Ghent, Belgium) to confirm the presence of the desired mutations. The obtained amplicons were loaded onto the ABI3100 Genetic Analyzer (Applera, Nieuwekerk a/d Issel, The Netherlands) after which the sequences were analyzed using the Geneious Pro 5.5.6 software.

The oligonucleotide sequences of the sequencing primers, of which some have been published before [31], are listed in Additional file 2: Table S1.

### Microscopic evaluation of syncytium formation and eGFP expression

Hundred thousand C8166 cells were seeded into wells of a 96-well plate and were infected with 4 ng p24 of WT or gp41 *N*-glycan*-*deleted HIV-1_NL4.3_, in a total volume of 200 μl per well. Three days post infection, giant cell formation and eGFP expression was visualized using the FLoid Cell Imaging System (Life Technologies, Ghent, Belgium).

### Quantitative determination of the infectivity potential of the mutant gp41 virus strains

Two approaches were used to quantitatively analyze virus infectivity.

One experiment was performed as described previously [11]. Briefly, 10^6^ C8166 cells were brought into wells of a 24-well plate and were infected with 40 ng p24 of WT or mutant gp41 HIV-1_NL4.3_ on day 0. By adding RPMI-1640 culture medium it was ascertained that every well contained a total volume of 2 ml. Every day, for 7 subsequent days, samples were harvested, fixed with 3% formaldehyde and stored at 4°C for further analysis. At the end of the incubation period, the percentage of infected cells was quantified for all samples by the analysis of eGFP expression with a FACS CantoII flow cytometer (BD biosciences, Erembodegem, Belgium). The data were analysed by FACS Diva Software (BD biosciences).

Alternatively, the viral infectivity of several WT and mutant gp41 N616Q virus strains was compared using C8166 and U87.CD4.CXCR4.CCR5 cells. Therefore, 30,000 C8166 and 7,000 U87.CD4.CXCR4.CCR5 cells were seeded in a 96 well plate. The C8166 cells were immediately exposed to 2,500, 1,250 or 625 pg p24 of the different virus strains. After 3 days, the C8166 cells were fixed in 3% formaldehyde and eGFP expression was analyzed using flow cytometry (FACS CantoII, BD biosciences). For the U87.CD4.CXCR4.CCR5 cells, infection was initiated 1 day after cell seeding and was evaluated microscopically (FLoid Cell Imaging System, Life Technologies) and by flow cytometry (FACS CantoII, BD biosciences) after 5 days.

### Quantification of mutant gp41 virus binding to sCD4

The experiment was performed as published before, although some adaptations were made [32]. Briefly, 8-well maxisorp strips (Nunc, Haasrode, Belgium) were coated for 2 hours at room temperature with 0.5 μg of soluble CD4 (Sino Biological Inc, Ghent, Belgium) in 50 mM carbonate buffer (pH 9.6). After washing with wash buffer (PBS supplemented with 0.05% Tween 20), the wells were blocked for 1 hour at room temperature using PBS pH 7.4 supplemented with 0.05% Tween 20 and 2% milk powder (= blocking buffer). After a next round of washing, approximately 48 ng p24 of virus lysed using 10% Triton*-*X100 in blocking buffer was added to the wells for 1 hour at 37°C. After washing, the wells were incubated with the primary sheep antibody directed against gp120 (D7324; Aalto Bio Reagents, Dublin, Ireland) in blocking buffer during 1 hour at 37°C. Afterwards, the wells were washed again and incubated for 1 hour at 37°C with the secondary antibody labelled with alkaline phosphatase in blocking buffer. Substrate buffer (1 mg/ml p-nitrophenyl phosphate (Sigma) dissolved in 10% diethanolamine, pH 9.8, with 0.5 MgCl_2_) was added to the wells after sufficient washings. Following 30 minutes incubation, the absorbance at 405 nm was determined using the Safire 2 microtiter plate reader (Tecan, Mechelen, Belgium).

### Western blot analysis to quantify env incorporation in the viral particles

The expression and incorporation levels of the envelope glycoproteins gp120 and gp41 in WT and mutant gp41 virus particles were determined by Western blot (WB) analysis as described previously [32]. 50 ng of p24 virus was loaded onto a 4-12% Bis-Tris PAGE gel (Invitrogen). After blotting, gp120, gp41 and p24 were detected using the primary antibodies PAI-7218 (Thermo Scientific, Erembodegem, Belgium), C8 and AB9044 (Abcam, Antwerp, Belgium), respectively. The monoclonal antibody directed to gp41, C8, was derived from HIV-1 gp41 Hybridoma (Chessie 8) cells and obtained through the NIH AIDS Reagent Program, Division of AIDS, NIAID, NIH, from Dr George Lewis [33]. Visualization was achieved by using secondary, horseradish peroxidase-labelled antibodies (Santa Cruz Biotechnology, Boechout, Belgium) and the SuperSignal West Pico Chemiluminescent Substrate Kit (Thermo Scientific). Imaging was performed using the BioRad ChemiDoc MP Imager (BioRad, Nazareth Eke, Belgium). The ImageJ software was used for quantification of the WB data. MagicMark XP Western protein standard (Novex) was used to confirm the molecular weight of gp120, gp41 and p24.

### Analysis of gp120 expression on the cell membrane of HIV-transfected HEK293T cells using flow cytometry

This experiment was performed as described earlier [34]. HEK293T cells were transfected to produce WT or mutant HIV-1_NL4.3_. Two or three days post transfection, the cells were stained to detect surface gp120 using the monoclonal antibody 2G12 and a secundary antibody labelled with Alexa Fluor 647 (Invitrogen). The cells were analysed using the FACSCanto II flow cytometer (BD Biosciences).

### Capture of HIV-1_NL4.3_ particles by Raji/DC-SIGN cells and the subsequent transmission of DC-SIGN-captured virions to C8166 cells

Wild-type and gp41 mutant HIV-1_NL4.3_ virus strains (50,000, 25,000 and 12,500 pg p24) were incubated with 10^6^ Raji/DC-SIGN cells for 1 h at 37°C in a volume of 1 ml, after which unbound virions were removed by thorough washings. The cells were resuspended in 1 ml of fresh culture medium and the amount of captured virions was determined using a p24 ELISA (PerkinElmer, Boston, MA). In parallel, 2x10^5^ virus-captured Raji/DC-SIGN cells were brought into contact with 2x10^5^ C8166 cells in a volume of 1 ml, resulting in the transmission of virions from the Raji/DC-SIGN cells to the C8166 cells and the subsequent infection of the C8166 cells. After 72 h of cocultivation, the formation of syncytia was evaluated microscopically and the amount of virus produced by the C8166 cells was determined by a p24 ELISA (PerkinElmer). The measured viral load was used as a parameter for the efficiency of transmission of virus from Raji/DC-SIGN cells to C8166 cells.

### Studying the binding kinetics of CBAs to gp120 and gp41

The SPR technology was used to study the interaction of a selection of CBAs (including 2G12) with gp120 and gp41. Therefore, gp120, gp41 and human serum albumin (HSA) were covalently bound to the surface of a CM4 chip (GE Healthcare, Uppsala, Sweden), at densities of approximately 115, 45 and 71 RU, using standard amine coupling. These low densities of immobilized ligands are necessary to prevent rebinding during the dissociation phase. HSA was used as a negative control, to take into account random protein*-*protein interactions. The interaction studies were performed using the Biacore T200 instrument (GE Healthcare), at a temperature of 25°C and with HBS-P (10 mM HEPES, 150 mM NaCl and 0.05% surfactant P20; pH 7.4) supplemented with 10 mM CaCl_2_ as running buffer. For each compound, a two-fold serial dilution was used with the highest CBA concentrations being 10 nM for HHA, 80 nM for UDA, 80 nM for AH and 500 nM for 2G12. Association was monitored during a 2 minute injection phase, at an injection rate of 30 μl/min. Afterwards, dissociation was monitored during 5 minutes. Residual bound analyte was eliminated by a regeneration step using 50 mM NaOH (in the case of HHA, UDA and AH) or Glycine-HCl pH 1.5 (in the case of 2G12) during 10 seconds at a rate of 30 μl/min. The obtained sensorgrams were used to perform a kinetic analysis, based on the 1:1 Langmuir binding model (GE Healthcare), resulting in K_D_ values for the studied CBA-(glyco)protein interactions.

### Determination of the sensitivity of the mutant gp41 virus strains for CBAs

Thirty thousand C8166 cells were brought into wells of a 96-well plate, in a total volume of 200 μl. The test compounds (HHA, UDA, AH, 2G12, PRM-S, T-20 and AMD3100) were added as a 5-fold serial dilution with the highest concentrations of 0.2, 2.3, 0.3, 0.2, 60, 3.3, 0.3 μM, respectively. WT or mutant HIV-1_NL4.3_ were added at viral loads resulting in approximately 10% eGFP positive cells at 72 hours post infection. After incubation at 37°C for 72 hours, the cells were fixed in 3% formaldehyde and analysed using the FACS CantoII flow cytometer (BD biosciences) to quantify eGFP expression as a measurement of viral infectivity. The 50%-effective concentration (EC_50_) was calculated as the compound concentration required to suppress viral infection by 50%.

### Statistics

A Student’s t-test was used to validate the significance of the data. A *p* value <0.05 was considered as significant.

## Additional files

Additional file 1: Figure S1. Original Western blot images used to generate Figure 5A. Images generated using Image Lab (Biorad) after an exposure of 8 s **(A)**, 32 s **(B)** and 82 s **(C)**. Image **A** was used for the visualization of p24, image **B** was used for the visualization of gp120, Image **C** was used for the visualization of gp41. M; MagicMark XP Western protein standard (Novex). Additional file 2: Table S1. Oligonucleotide sequences of the primers used for cloning, site-directed mutagenesis and sequencing of gp41.

## Abbreviations

AH: Actinohivin
CBA: Carbohydrate-binding agent
EC_50_: 50% effective concentration
eGFP: Enhanced green fluorescent protein
ELISA: Enzyme-linked immunosorbent assay
GNA: *Galanthus nivalis* agglutinin
HHA: *Hippeastrum* hybrid agglutinin
HR: Heptad repeat
K_D_: Dissociation constant
PRM-S: Pradimicin-S
sCD4: Soluble CD4
UDA: *Urtica dioica* agglutinin
SPR: surface plasmon resonance
T-20: enfuvirtide
WB: Western blot
WT: Wild-Type
WT∆Env: WT virus lacking envelope glycoprotein expression
