# Supplementary figures and images for: The role of N-glycans of HIV-1 gp41 in virus infectivity and susceptibility to the suppressive effects of carbohydrate-binding agents
Source: Retrovirology. 2014 Dec 11;11:107. doi: 10.1186/s12977-014-0107-7 (PMC4269863; doi:10.1186/s12977-014-0107-7)

**A**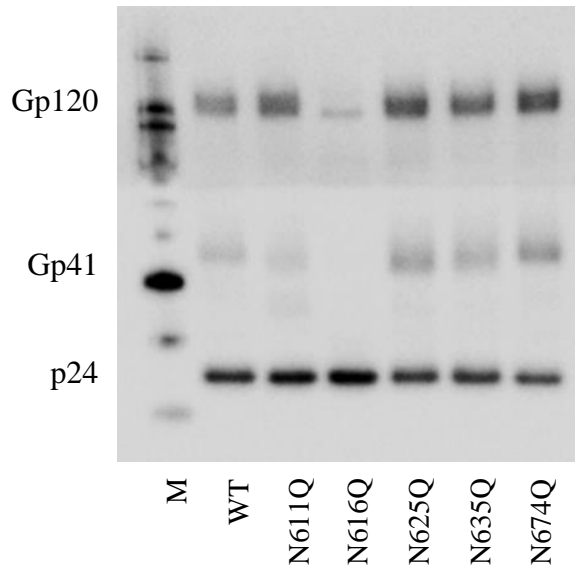**B**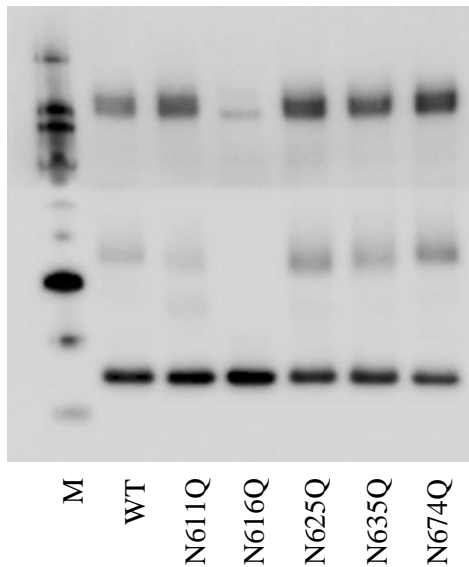**C**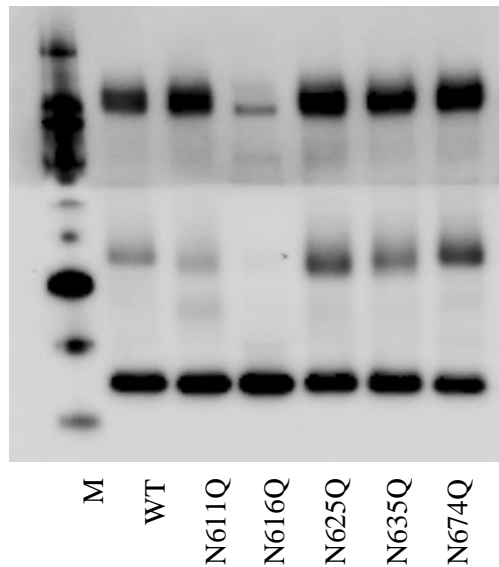

Supplement: Additional file 1: Figure S1. — Original Western blot images used to generate Figure 5A. Images generated using Image Lab (Biorad) after an exposure of 8 s (A), 32 s (B) and 82 s (C). Image A was used for the visualization of p24, image B was used for the visualization of gp120, Image C was used for the visualization of gp41. M; MagicMark XP Western protein standard (Novex). [file 12977_2014_107_MOESM1_ESM.pdf]
