# Supplementary material for: The role of N-glycans of HIV-1 gp41 in virus infectivity and susceptibility to the suppressive effects of carbohydrate-binding agents
Source: Retrovirology. 2014 Dec 11;11:107. doi: 10.1186/s12977-014-0107-7 (PMC4269863; doi:10.1186/s12977-014-0107-7)
Supplement: Additional file 2: Table S1. — Oligonucleotide sequences of the primers used for cloning, site-directed mutagenesis and sequencing of gp41. [file 12977_2014_107_MOESM2_ESM.pdf]

Table S1. Oligonucleotide sequences of the primers used for cloning, site-directed mutagenesis and sequencing of gp41.

Primer	Nucleotide sequence	Description
EnvB_EcoRI	GTCAGAGAATTCAGAAAGAGCAGAAGACAGTGGCAA TGA	Forward cloning primer
HIV8726-R_XhoI	CGT CGT CTC GAG TTG TAC TAC TTC TAT AAC CCT ATC TGT	Reverse cloning primer
Mut_N611Q_F	GCT GTG CCT TGG CAA GCT AGT TGG AG	Forward mutagenesis primer for N611Q
Mut_N611Q_R	CTC CAA CTA GCT TGC CAA GGC ACA GC	Reverse mutagenesis primer for N611Q
Mut_N616Q_F	GCT AGT TGG AGT CAA AAA TCT CTG GAA C	Forward mutagenesis primer for N616Q
Mut_N616Q_R	GTT CCA GAG ATT TTT GAC TCC AAC TAG C	Reverse mutagenesis primer for N616Q
Mut_N625Q_F	CAG ATT TGG AAT CAG ATG ACC TGG ATG G	Forward mutagenesis primer for N625Q
Mut_N625Q_R	CCA TCC AGG TCA TCT GAT TCC AAA TCT G	Reverse mutagenesis primer for N625Q
Mut_N637Q_F	CAG AGA AAT TAA CCA ATA CAC AAG CTT AAT AC	Forward mutagenesis primer for N637Q
Mut_N637Q_R	GTA TTA AGC TTG TGT ATT GGT TAA TTT CTC TG	Reverse mutagenesis primer for N637Q
Mut_N674Q_F	GTT TGT GGA ATT GGT TTC AGA TAA CAA ATT GGC TGT G	Forward mutagenesis primer for N674Q
Mut_N674Q_R	CAC AGC CAA TTT GTT ATC TGA AAC CAA TTC CAC AAA C	Reverse mutagenesis primer for N674Q
Mut_N672D_F	GTT TGT GGA ATT GGT TTGATA TAA CAA ATT GGC TGT G	Forward mutagenesis primer for N674D
Mut_N672D_R	CAC AGC CAA TTT GTT ATA TCA AAC CAA TTC CAC AAA C	Reverse mutagenesis primer for N674D
Mut_N616Q_IIIB_F	GCT AGT TGG AGT CAG AAA TCT CTG GAA C	Forward mutagenesis primer for N616Q in gp41 of strain III_B
Mut_N616Q_IIIB_R	GTT CCA GAG ATT TCT GAC TCC AAC TAG C	Reverse mutagenesis primer for N616Q in gp41 of strain III_B
Mut_N616Q_ADA_F	GCT AGT TGG AGT CAG AAA TCC TAC AGT CAG	Forward mutagenesis primer for N616Q in gp41 of strain ADA
Mut_N616Q_ADA_R	CTG ACT GTA GGA TTT CTG ACT CCA ACT AGC	Reverse mutagenesis primer for N616Q in gp41 of strain ADA
Mut_N616Q_HE_F	CTA GTT GGA GTC AGA AAT CTC TGC	Forward mutagenesis primer for N616Q in gp41 of strain HE
Mut_N616Q_HE_R	GCA GAG ATT TCT GAC TCC AAC TAG	Reverse mutagenesis primer for N616Q in gp41 of strain HE
AV324 [1]	GGCAAAGAGAAGAGTGGT	Forward sequencing primer
AV329 [1]	GTCCCAGAAGTTCCACA	Reverse sequencing primer

Primers AV324 and AV329 have been published before in:

1. Van Laethem K, Schrooten Y, Lemey P, Van Wijngaerden E, De Wit S, Van Ranst M, Vandamme AM: **A genotypic resistance assay for the detection of drug resistance in the human immunodeficiency virus type 1 envelope gene.** *J Virol Methods* 2005, **123**:25-34.
